# Administration of Single or Repeated Doses of CDCs in a Swine Model of Reperfused Myocardial Infarction: Magnetic Resonance and Proteomics Evaluation

**DOI:** 10.3390/ijms262311294

**Published:** 2025-11-22

**Authors:** María Ángeles de Pedro, Claudia Báez-Díaz, Inmaculada Jorge, Fátima Vázquez-Lopez, Axiel Torrescusa-Bermejo, Beatriz Martinez-Fernandez, María Pulido, Esther López, Jesús Vázquez, Francisco M. Sánchez-Margallo, Veronica Crisostomo

**Affiliations:** 1 Centro de Cirugía de Mínima Invasión Jesús Usón, 10071 Cáceres, Spain; 2 Red Española de Terapias Avanzadas RICORS-TERAV, 28029 Madrid, Spain; 3 Centro Nacional de Investigaciones Cardiovasculares, 28029 Madrid, Spain; 4 CIBER de Enfermedades Cardiovasculares (CIBERCV), 28029 Madrid, Spain; 5 Micros Veterinaria, 24007 León, Spain

**Keywords:** experimental myocardial infarction, cardiosphere-derived cells, CDCs, proteomics

## Abstract

Some studies report better outcomes in cell therapy for myocardial infarction (MI) with repeated administrations. We aimed to elucidate the potential differences in terms of cardiac function and protein expression after one or three doses of cardiosphere-derived cells (CDCs) in a porcine MI model. CDCs were isolated from swine cardiac explants, cultured in cardiomyocyte growth medium (CGM), and prepared for administration. Pigs surviving a 90 min balloon occlusion of the mid-left anterior descending coronary artery (LAD) were randomly allocated to receive vehicle (CON), one (D1), or three (D3) doses of 30 × 10^6^ CDCs via the infarct-related coronary artery. Cardiac function was assessed with magnetic resonance at baseline and 10 weeks. Programmed electrical stimulation to study arrhythmogenicity was performed at 10 weeks. High-throughput quantitative proteomic analysis of infarcted tissue was performed to identify biological processes based on protein abundance changes between groups. No significant differences were found between the three groups for any cardiac function parameter at 10 weeks. No increase in ventricular tachycardia inducibility was seen in treated groups. However, gene ontology and topological analyses revealed potentially beneficial molecular adaptations. Upregulation of GYS1, AGL, and GBE1 indicated an increase in glycogen biosynthesis and energy availability, while an increase in ANK2, along with hub proteins ALB and TRAP1, suggested cardioprotective effects. Furthermore, the increase in remodeling-related proteins, including EPHA4, PODN, and ALPK3, pointed to favorable structural adaptation following infarction. In conclusion, the intracoronary administration of single or repeated doses of 30 × 10^6^ CDCs to a porcine reperfused MI model shows only slight differential improvement in both cardiac function and protein profile in this experimental setting, thus presenting limited translational potential.

## 1. Introduction

Ischemia-reperfusion (IR) injury following myocardial infarction (MI) therapy causes an increase in the incidence of heart failure (HF), an entity with poor prognosis and that is associated with low quality of life, high healthcare costs, and a high mortality rate. Therefore, the development of new therapeutic alternatives is needed. Regenerative therapies, including cell therapy, may be advantageous for these patients. Initial interest in cardiac cell therapies was extremely high, as were expectations. Cell therapy implementation, as has been proposed in the last two decades, has clearly failed to deliver [1]. These disappointing results can be attributed to many reasons, and probably not all of them have been identified to date. Some of them may include the cardiac milieu, extremely hostile to the transplanted cells that precludes survival in the acute phase, the use of rodent models in preclinical trials (what has been termed “the translational axis” [2]), or a persistent failure to recognize that a single therapeutic dose is incapable of preventing/reverting a complex multifactorial process that has taken years or decades to develop [3]. In this line, several studies in cell therapy for MI suggest that outcomes could be improved with repeated administration [3,4,5,6,7,8,9,10,11,12,13,14,15,16]. Because of the short-lived paracrine actions of an administered single-dose cell, and even shorter cell engraftment and survival, it may be naïve to expect long-lasting clinical improvement with a single-dose protocol [17]. Beneficial results in repeated cell administration have been hypothesized to be based on immunomodulation elicited by paracrine factors released by the cells, since engraftment is consistently low or negligible, but mechanistic understanding of these effects is still lacking [18].

Among potential candidate cells, cardiac-derived cell products, including cardiosphere-derived cells (CDCs), cardiac progenitor cells (CPCs), or c-kit positive cells, have shown promise in myocardial repair after acute myocardial infarction (AMI), but single-dose trials have yielded moderate results at best. Experience with repeated administration of these cells is mostly limited to rodents [5,7,9,11,13,14,15], without any studies to date reporting their repeated use in large animal models. The use of representative preclinical models is mandatory prior to translating experimental findings into clinical practice. As such, the swine reperfused infarction has been repeatedly validated for this purpose [19,20]. As reported in other species, single-dose trials with cardiac-derived cell products in pigs have yielded modest functional results in the best of cases [21,22,23], but experimental evidence accumulated in other species [5,7,9,11,13,14,15] or with different cell types [4,10,24] suggests that repeated administration could improve results.

To explore the usefulness of a therapeutic intervention, not only clinical-grade gold standard technologies, i.e., cardiac magnetic resonance (CMR), and representative animal models such as swine are necessary but a deep understanding of why a therapy works also needs to be explored. Proteomic studies have the potential to provide insights into the underlying molecular mechanism via the identification of protein abundance changes across different pathways, thus complementing functional and safety data derived from other techniques such as CMR or programmed electrical stimulation (PES).

In this context, we aimed to elucidate the potential differences in terms of cardiac function, arrhythmogenicity, and protein abundance after one or three intracoronary (IC) CDC doses in an experimental model of severely impaired heart function secondary to MI in swine to assess differential safety and efficacy, as well as to identify potential molecular markers of therapeutic effect.

## 2. Results

A total of 30 large white (LW) pigs, weighing 40.1 ± 1.1 kg, were included in the study. Of these, four animals (13.3%) died during infarct creation. At one week after infarction, baseline CMR was performed, and a further two animals were excluded due to left ventricular ejection fraction (LVEF) > 30% (33% and 35%); therefore, eight animals were randomized to each group.

### 2.1. Safety of Single or Repeated CDC Administration

All animals received the IC therapy in the left anterior descending coronary artery (LAD) in the absence of complications or ECG alterations. No changes to the ECG or postoperative fibrillation or deaths were observed in any animal, irrespective of the group. Similarly, there were no differences in TnI or CRP when comparing before or after each injection, independently of the dose, nor were there any differences between treated and control groups after each injection or at the end-study measurement (Table 1).

As to long-term safety, no deaths occurred during follow-up. End-study programmed electrical stimulation was performed to attempt ventricular tachycardia induction and resulted in a similar rate of inducibility between groups (*p* = 0.83), with no increase in arrhythmogenesis associated with the administered therapy. Pathological examination did not reveal any instances of abnormal tissue growth or persistent inflammation.

### 2.2. Efficacy of Single or Repeated CDC Administration

Cardiac function parameters and infarct size, as measured in the CMR examinations performed at 1 and 10 weeks, are shown in Table 2. No statistically significant differences were found between the three groups in any parameter at 10 weeks.

**Figure 1:**
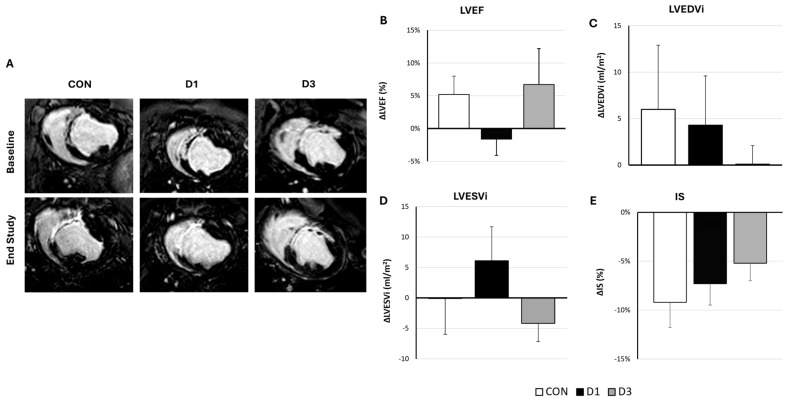
Evolution of cardiac function parameters after the administration of vehicle, one, or three CDCs doses in infarcted swine. (**A**) Representative delayed enhanced images obtained before the first administration and at end-study (10 weeks) from the three experimental groups (CON: Control, D1: one CDCs dose, D3: three CDCs doses). (**B**–**E**) Treatment effects (defined as the difference between pre-injection and 10-week values) in cardiac function parameters as measured with cardiac magnetic resonance. No significant differences were evidenced between groups in any parameter (*p* > 0.05). Error bars represent standard error of the mean; (**B**) Left ventricular ejection fraction (LVEF); (**C**) Left ventricular end-diastolic volume indexed to body surface area (LVEDVi); (**D**) Left ventricular end-systolic volume indexed to body surface area (LVESVi); and (**E**) Infarct size (IS).

As shown in Figure 1, a trend towards better outcomes in D3 over time, with improved treatment effects, was evidenced in all cardiac function parameters measured (∆LVEF was 5.2 (2.8) % vs. −1.6 (2.5) % vs. 6.7 (5.5) %, ∆LVESVi was −0.1 (5.9) mL/m^2^ vs. 6.1 (5.6) mL/m^2^ vs. −4.2 (3.0) mL/m^2^, and ∆LVEDVi was 6.0 (6.9) mL/m^2^ vs. 4.3 (5.3) mL/m^2^ vs. 0.1 (2.0) mL/m^2^ in CON, D1, and D3 groups, respectively).

**Figure 2:**
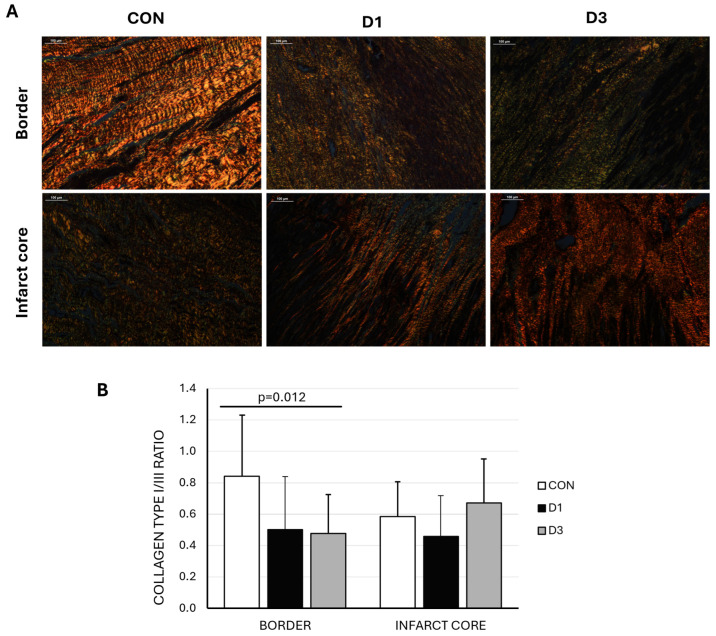
(**A**) Representative polarized light microscopy images of picrosirius red staining in border and infarct core samples from swine belonging to the three experimental groups (CON: control, D1: one CDC dose, D3: three CDC doses). Type I collagen fibrils are shown in red/yellow and type III in green (scale bar = 100 μm). (**B**) Collagen I/III ratio was significantly greater in the border zone of CON animals.

Pathological examinations showed transmural fibrous scars of similar sizes and maturation as reflected in the hematoxylin eosin (HE) and Masson’s trichrome (MT) staining, in the absence of evident pathological differences (Supplementary Figure S1). The study of collagen types present at the infarct and border zone revealed a higher type I/III collagen ratio at the border zone in the control group compared to both D1 and D3 (*p* = 0.012), indicating increased stiffness in this group (Figure 2).

### 2.3. Proteomic Assessment of Cardiac Response to CDC Therapy

**Figure 3:**
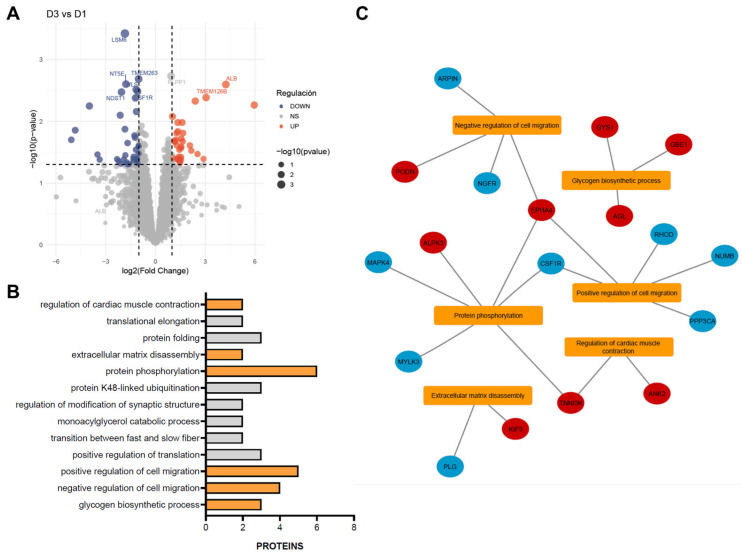
Functional enrichment of differentially abundant proteins (DAPs) between single- and triple-dose CDC treatments. (**A**) Volcano plot showing differential protein abundance between triple- and single-dose treatments. Red dots represent significantly increased proteins, and blue dots represent significantly decreased proteins (thresholds: *p* < 0.05 and |log_2_ fold change| > 1). The top 10 most significantly altered proteins are labeled on the plot. (**B**) The most significantly enriched gene ontology (GO) biological processes are shown, with terms related to myocardial infarction highlighted in orange. (**C**) Interaction network linking the enriched GO terms with their corresponding DAPs. Red nodes indicate upregulated proteins, and blue nodes indicate downregulated proteins.

As the functional improvements observed were modest, we sought to investigate the underlying molecular mechanisms by performing a proteomic analysis of infarcted myocardium following single or repeated CDC administrations. Differential protein abundance was assessed using a volcano plot, which highlighted proteins exhibiting significant changes between control and treatment groups. A total of 121 differentially abundant proteins (DAPs) were identified across one and three treatment doses, showing a nearly balanced distribution of upregulated (51.24%) and downregulated (48.76%) proteins (Figure 3A). These DAPs were subsequently subjected to functional enrichment analysis to identify the biological processes and pathways modulated by CDC treatment.

Functional enrichment analysis revealed 13 significantly enriched biological processes (BPs) (Figure 3B), 6 of which are directly implicated in MI, ranked by statistical significance (*p*-value). The *glycogen biosynthetic process* (GO:0005978) was represented by GYS1, AGL, and GBE1, all showing increased abundance after three doses, suggesting enhanced glycogen storage and improved energy availability in cardiac tissue. *Regulation of cell migration* was represented in both *negative* (GO:0030336) and *positive* (GO:0030335) processes. The negative regulation group included EPHA4, PODN, NGFR, and ARPIN, displaying a balanced pattern of up- and downregulation. The positive regulation group comprised EPHA4, NUMB, CSF1R, PPP3CA, and RHOD, with all proteins downregulated except for EPHA4. *Protein phosphorylation* (GO:0006468), a key mechanism in cardiac signaling, showed upregulation of EPHA4, ALPK3, and TNNI3K and downregulation of CSF1R, MYLK3, and MAPK4. *Extracellular matrix disassembly* (GO:0022617) involved an increase in KIF9 and a decrease in PLG. Finally, *regulation of cardiac muscle contraction* (GO:0055117) included ANK2 and TNNI3K, both upregulated (Figure 3C).

Complementary KEGG pathway enrichment analysis identified only three significant categories: metabolic pathways, the most significant; oxytocin signaling pathway; and biosynthesis of amino acids (Supplementary Figure S2). These results reinforce the metabolic and biosynthetic shifts observed in the GO analysis.

**Figure 4:**
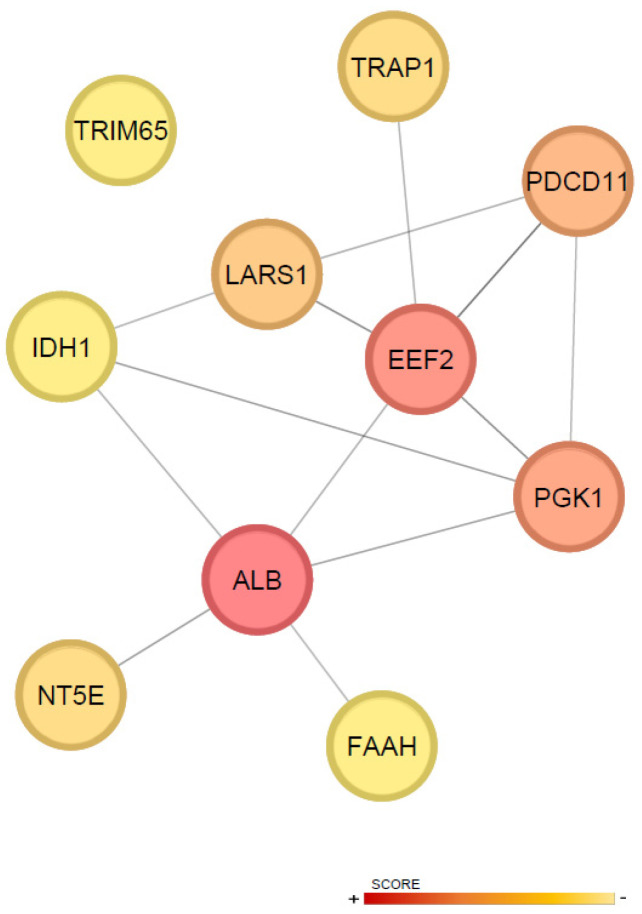
Topological characterization of hub proteins. The top ten hub proteins were identified based on centrality scores, highlighting key nodes in the network. Nodes with higher centrality are colored red, indicating greater importance, while nodes with lower centrality are colored yellow.

Moreover, topological analysis of the DAPs within the protein–protein interaction (PPI) network, using CytoHubba, identified the top ten hub proteins ranked by centrality score: ALB, EEF2, PGK1, PDCD11, LARS1, TRAP1, NT5E, FAAH, TRIM65, and IDH1 (Figure 4). These hubs likely play key regulatory roles in the molecular effect of the treatments.

## 3. Discussion

Stem cell therapy for ischemic cardiac diseases has been studied since the early 2000s with limited success [25,26], and a myriad of different approaches have been assayed to improve the results. Among these, repeated cell dosing appears to be promising, with a paradigm change having been proposed [3]. Since large animal studies are considered mandatory prior to human trials, in the present work, we studied the IC administration of single or repeated doses of 30 × 10^6^ CDCs in a porcine reperfused MI model of severely impaired cardiac function, attempting to recapitulate rodent results. However, our clinically relevant study showed only slight, not significant, differential improvement in both cardiac function and protein expression in this experimental setting, which points to limited direct translational potential.

To optimize our chances of obtaining clinically relevant results and following the recommendations outlined by opinion leaders in the field [27], we used relevant, clinical-grade technologies for our study, such as CMR and PES. Unexpectedly, we failed to identify a clear improvement trend despite all the available literature pointing to much greater effects of repeated dosing. In a recent editorial [18] breaking down the results of Tang et al. [4], it is stated that while three intravenous doses of mesenchymal stromal cells (MSCs) are better than none in a swine model of chronic MI, there is no statistically significant difference between one and three doses. In line with this, we did not find any significant results in cardiac function when comparing CON, D1, and D3. As is commonly seen with cell therapy studies, the possible reasons for this discrepancy with previously reported results may be found in the variety of stem cell types used (only one prior study used CDCs [14], with most employing MSCs [4,8,9,10,12] or CPCs [5,7,13,15]); the different animal models studied (rodents are the most common species); and the timing of cell therapy. In this sense, most studies deal with a chronic infarction and administer the therapy in 4- to 6-week intervals [5,7,8,9,15]. This scenario is different from the one studied in our work, where we administer the therapy one, two, and three weeks after model induction, which probably translates into a hostile cardiac milieu that could affect cell survival and therefore greatly limit its effects.

Additionally, the delivery method may also explain the different results. We used the IC route to increase the translatability of our study, since this is a widespread technique that could be easily adopted into clinical practice. Only other prior study in pigs [24] used a transendocardial approach and administered skeletal myoblasts in a chronic infarction. They report cumulative functional improvement and decreased fibrosis in treated animals, with a collagen volume fraction significantly different in control compared to treated animals. Likewise, we did find a higher type I/III collagen ratio at the border zone in the control group compared to both D1 and D3, which in our case did not translate into clinical benefit. They do not report arrhythmogenesis, despite some concern remaining amongst the scientific community as to whether cell therapies could prove arrhythmogenic [28], and their use of a cell type that has been abandoned due to arrhythmia issues. To assuage these safety concerns, we performed an electrophysiological study prior to euthanasia, including provocative testing and attempted arrhythmia induction, and found no increase in inducible arrhythmia related to cell therapy administration in this study. This is a safety evaluation which is also instrumental in bridging the bench-to-bedside gap, as post-infarction ventricular tachycardias stem from heterogeneous tissue within the myocardial scar, a mixture of cells that could be obtained by promoting myocyte survival/regeneration via cell therapy. This absence of arrhythmogenicity is not surprising, since in a prior study we have demonstrated that cardiosphere-derived cell secretome (S-CDC) can protect from arrhythmia in a very similar experimental setting [29].

Despite the paucity of preclinical studies, some human trials have been conducted with repeated administration using different cell types and delivery strategies, generally suggesting a positive outcome. IC injection of autologous peripheral blood stem cells (PBSCs) in patients with recent large AMI was reported to significantly improve LVEF in a pilot study [30], with a mean 8.9% improvement in the repeated administration group compared to 5.6% in single administration and 1.6% in the control group. Yao et al. [31] included patients with large myocardial infarction and substantial LVEF impairment (<30%) who were enrolled within one week of reperfusion and treated with single or repeated IC administration of autologous bone marrow mononuclear cells (BM-MNCs), reporting a significant cumulative improvement in LVEF after 12 months (2.9% versus a 7.2% and 11.7% change in LVEF in CON in one- and two-dose groups, respectively). Conversely, no effect on LVEF was shown using these cells in the setting of congestive heart failure when delivered IC [32], nor in the setting of refractory angina via the intramyocardial route [33], despite improved myocardial perfusion and decreased anginal complaints in this last study. In the only study using allogeneic cell sources so far, Attar et al. [16] recently administered one or two doses of 10^7^ Wharton’s jelly MSCs, reporting a 7.45% improvement in LVEF at 6 months in patients receiving two doses. Importantly, an inclusion criterium in this study was also a decreased LVEF (<40% as measured with echo). Left ventricular systolic function is a widely recognized prognostic measure of cardiovascular risk, representing an established predictor of adverse cardiovascular outcomes, especially in patients presenting with moderate to high reduction in LVEF [34]. As detailed above, only those studies enrolling patients with low LVEF have been able to report significant improvements, which is in line with prior results that have provided evidence that the effect of cell therapy is greater when cardiac function is more compromised [35]. Accordingly, to enter our study, infarcted animals were required to have an LVEF below 30%, representing severe cardiac dysfunction. In patients, LVEF < 30% is considered advanced heart failure, and mortality is high [34]. In our otherwise healthy swine, no mortality was evidenced beyond model creation, and all animals that survived induction reached the end-study timepoint. While selecting this severely impaired model should have resulted in a greater LVEF improvement, we failed to demonstrate it despite numerical results being consistently better in D3 animals (lower ventricular volumes and greater LVEF).

While this lack of direct efficacy is discouraging, it does not imply that these studies should be dismissed. Instead, it highlights the need to delve deeper into the possible reasons behind these findings. Such insights will help inform future research efforts in the field.

In this sense, it is important to consider that molecular changes may precede changes in heart function and structure and, as such, could be used as biomarkers for cardiac function prediction, response to therapy (or absence thereof), or disease progression. We, therefore, used comprehensive proteomic analyses to evaluate changes between groups. Additionally, such proteomic approaches can provide insights into the underlying mechanisms explaining why our therapy did not achieve the expected functional improvements observed in previous studies.

The analysis of the differential effect between the administration of one versus three doses of CDCs in the treatment of MI revealed proteomic changes suggesting a modest but potentially beneficial adaptation in the pathophysiology of ischemia–reperfusion injury. Among the most relevant findings was the upregulation of enzymes involved in glycogen biosynthesis (GYS1, AGL, GBE1), indicating an enhanced myocardial capacity to establish energy reserves. During MI, these augmented glycogen stores may transiently delay functional deterioration and mitigate tissue damage by providing energy during ischemic periods, thereby exerting a cardioprotective effect [36]. This observation suggests that treatment with three doses may confer a metabolic advantage to the myocardium, improving its tolerance to ischemic injury.

Another relevant finding was the modulation of extracellular matrix (ECM) disassembly processes and their potential impact on cardiac repair and scar formation. Deficiency in KLF9 has been associated with protection against adverse remodeling and post-MI inflammation through inhibition of NF-κB and MAPK activation in macrophages, thereby suppressing detrimental inflammatory responses [37]. Thus, reduced expression of this protein may favor adequate remodeling. Conversely, the downregulation of PLG, which has also been linked to ECM remodeling during infarct healing, may compromise scar formation and increase the risk of maladaptive remodeling. However, in a mouse model of MI, Plg−/− animals exhibited only minimal impairment of cardiac function compared with wild-type counterparts [38].

Other regulated factors also pointed to significant changes in cell migration processes essential for proper repair upon repeated dosing. Overexpression of EPHA4 suggests enhanced angiogenesis and cell migration, processes critical for granulation tissue formation and restoration of blood flow in the infarcted area [39]. By contrast, EphA4 knockout rats display atrial hypertrophy and electrocardiographic abnormalities [40]. Similarly, upregulation of the ECM protein PODN may contribute to favorable remodeling by inhibiting excessive smooth muscle cell proliferation and limiting fibrosis without inducing fibrinolysis, supporting its potential protective role following cardiac injury [41]. Not all dose-dependent changes were advantageous, however. The downregulation of CSF1R is particularly noteworthy, as this receptor is essential for macrophage survival and activation, cells critical for inflammation resolution and tissue repair [42]. Its reduction could hinder necrotic tissue clearance and the generation of reparative signals.

Phosphorylation pathway modulation further indicated a dual role mediated by kinases. ALPK3 plays a critical role in cardiac differentiation, cardiomyocyte proliferation, and sarcomeric proteostasis [43]. Loss of ALPK3 has been associated with contractile dysfunction and dilated cardiomyopathy in both murine and human models [44], whereas truncating variants are linked to hypertrophic cardiomyopathy [43]. Complementarily, downregulation of MYLK3, a kinase essential for cardiac contraction, may weaken myocardial contractility [45].

Regarding regulation of myocardial contractility, increases in ANK2 and TNNI3K were observed. Li et al. identified the ATP-sensitive K+ channel–ankyrin-B association as a cardioprotective mechanism against acute ischemia [46], whereas ANK2 loss-of-function variants are associated with ankyrin-B syndrome, a complex and potentially lethal cardiac phenotype [47]. TNNI3K, on the other hand, plays a multifaceted role in MI and often acts as a detrimental factor by enlarging infarct size, promoting oxidative stress, and inducing cell death. Mouse models with elevated TNNI3K expression exhibit worsened cardiac phenotypes, reduced recovery after ischemia/reperfusion, adverse remodeling, and slowed conduction [48]. In line with this, gain-of-function model, cardiomyocyte-specific deletion of TNNI3K resulted in smaller infarcts and lower production of injury markers compared with wild-type mice [49]. Nevertheless, some evidence suggests that TNNI3K overexpression may exert protective effects by reducing injury and improving cardiac function through suppression of p38/JNK-mediated apoptosis [50].

Consistent with GO analysis, topological algorithm-based prediction of the ten most significant differentially abundant proteins (hubs) within the network allowed the identification of key mechanisms favored by the three-dose regimen. This profile is characterized by convergence of pathways related to energy metabolism, apoptosis, modulation of the inflammatory response, and tissue repair mechanisms.

Among the hubs, serum albumin (ALB) exhibited the highest score. This protein has demonstrated prognostic value both in the acute MI phase and long-term follow-up, with low levels correlating with higher mortality and poorer functional outcomes [51]. From a metabolic and antioxidant perspective, the mitochondrial chaperone TRAP1 contributes to preserving mitochondrial function and reducing oxidative stress during ischemia–reperfusion, promoting cardiomyocyte survival; its upregulation is associated with cardioprotection [52].

In the regulation of apoptosis, the elongation factor eEF2 exhibits a bidirectional role. While the active form of eEF2 promotes protein synthesis and the expression of anti-apoptotic factors such as Bcl-2, its phosphorylation facilitates nuclear apoptosis, reflecting a delicate balance between cell survival and death [53].

Regarding inflammation, ecto-5′-nucleotidase NT5E/CD73 is critical for extracellular adenosine generation and the activation of anti-inflammatory, angiogenic, and reparative pathways. Its downregulation is associated with reduced adenosine production, increased immune infiltration, impaired angiogenesis, and adverse remodeling, leading to larger infarct size and enhanced fibrosis [54]. Conversely, fatty acid amide hydrolase (FAAH) modulates inflammation and extracellular matrix remodeling. Although repeated dosing increased the expression of this hub protein, FAAH deficiency has been associated with elevated macrophage infiltration, cardiomyocyte loss, and impaired ventricular function [55]. FAAH−/− mice exhibited increased inflammation, cardiomyocyte loss, more pronounced remodeling, and persistent scarring with left ventricular dysfunction compared with wild-type littermates [55].

Finally, in terms of tissue remodeling and repair, TRIM65 exhibits context-dependent behavior: appropriate expression attenuates hypertrophy and pathological remodeling by promoting autophagy and preserving mitochondrial function, whereas its downregulation enhances NLRP3 inflammasome activation, increases apoptosis, and leads to more severe fibrosis, contributing to poorer functional outcomes [56].

In addition, complementary KEGG pathway analysis reinforced these GO-based findings, highlighting overlapping metabolic and biosynthetic adaptations that may support tissue repair. The enrichment of metabolic pathways and biosynthesis of amino acids aligns with the observed upregulation of glycogen-related and energy-handling proteins, while oxytocin signaling may reflect additional cardioprotective and reparative mechanisms.

Overall, repeated administration of CDCs appears to enhance certain beneficial effects; however, these changes do not seem to translate into clear functional improvement. This may be partly explained by the fact that, at the proteomic level, our study specifically focused on the differential impact of administering one versus three doses of CDCs after myocardial infarction rather than providing an exhaustive characterization of the entire proteome—an undertaking that would require a dedicated study. Given the complexity of the infarcted myocardium, the processes identified here represent only a fraction of the broader adaptive landscape triggered by ischemic injury and subsequent repair. As a result, numerous unexamined mechanisms may be contributing to the lack of substantial functional recovery observed.

Taken as a whole, our results show that repeated CDC administration elicited potentially relevant proteomic adaptations, particularly in pathways related to post-infarction repair, yet these molecular shifts did not translate into measurable functional improvement. This disconnect suggests that the incremental benefits observed at the proteomic level are insufficient to drive meaningful changes in cardiac performance, thereby limiting the direct translational potential of this strategy.

## 4. Materials and Methods

This study was performed following the Directive 2010/63/EU of the European Parliament on the protection of animals used for scientific purposes, after obtaining approval from the Institutional Animal Care and Use Committee and Competent Authority (Extremadura Regional Government, EXP-20210629). The ARRIVE Guidelines for reporting experiments involving animals have been followed for reporting this experimental, randomized, blinded outcomes prospective study.

### 4.1. Isolation of CDCs and Therapy Preparation

CDCs were obtained from the stem cell bank of the Cell Therapy Unit at the Jesús Usón Minimally Invasive Surgery Centre. For CDC isolation, cardiac tissue explants from healthy LW pigs were mechanically dissociated and subjected to three sequential digestions with 0.2% trypsin (Lonza, Basel, Switzerland) and 0.2% collagenase IV (Sigma-Aldrich, St. Louis, MO, USA), as previously described [57]. CDC identity was confirmed by flow cytometry and their multipotent differentiation capacity toward adipogenic, chondrogenic, and osteogenic lineages, in accordance with the guidelines of the International Society for Cell Therapy [58]. Therapeutic preparations were administered in a blinded manner, with a standardized volume of 20 mL prepared irrespective of content and delivered using a blinded syringe to ensure procedural masking.

### 4.2. Swine Model of Reperfused Myocardial Infarction

Thirty female LW swine were included in the study. Animals were assigned a unique alphanumerical code and randomization and group allocation were performed by an independent researcher (a team member involved only in the preparation of the therapies). To minimize fatal arrhythmias during infarction, 400 mg oral amiodarone was administered daily from 5 days before infarct induction to 3 days after it. On each study day, animals were premedicated with an intramuscular (I.M.) combination of 20 mg/kg ketamine (Ketamidor 100 mg/mL, Richter Pharma AG, Wels, Austria) and 0.25 mg/kg of diazepam (Diazedor, VetViva Richter GmbH, Wels, Austria), and anesthesia was induced with 3 mg/kg intravenous 1% propofol (Propofol-Lipuro; B Braun Ltd., Melsungen, Germany) and maintained with inhaled sevoflurane (1.8–2% inspiratory fraction) as detailed elsewhere [29]. Postoperative analgesia was assured with I.M. buprenorphine (10 μg/kg/12 h) during the first 24 h combined with a fentanyl transdermal release patch (50 μg/h).

Animals were subjected to a 90 min occlusion of the left anterior descending coronary artery with an angioplasty balloon catheter inserted via a femoral approach, as previously described [23].

### 4.3. Treatment Protocols

One week after infarct induction, CMR was performed in surviving animals to assess cardiac function and infarct size. Subjects with infarct size >10% of the left ventricle and ejection fraction <30% were included in the study and randomly allocated (1:1:1) to one of three groups: CON (these subjects received 20 mL saline via the infarct-related artery); D1 (these subjects received 30 × 10^6^ CDCs in 20 mL saline on day 7 after infarct induction); and D3 (these subjects were administered three cell doses of 30 × 10^6^ CDCs in 20 mL saline on days 7, 14, and 21 after infarct induction).

Blood sampling was performed immediately before and 1 h after each intervention, as well as on the end-study day, to check for variations in CRP or TnI that could indicate deleterious effects of the administered therapy.

### 4.4. Therapy Administration

One, two, and three weeks after MI induction, surviving animals were blindly treated as per the groups’ distribution. Access to the LAD was established in anesthetized animals as described for infarct creation, a coronary angiogram obtained to assess flow in the LAD, and a 3 Fr microcatheter navigated to the level of the prior coronary occlusion. The total volume to be infused was administered manually at an injection rate of 1 mL/min without stop-flow conditions. A completion coronary angiogram was acquired 5 min after administration to assess coronary patency post-administration. The femoral sheath was removed, hemostasis of the puncture site achieved by manual compression, and the animals allowed to recover from anesthesia and transported back to the animal housing facility.

Immediate safety included the peri-procedural appearance of major adverse cardiac events such as death or ventricular fibrillation requiring cardioversion, ECG changes (ST segment, T wave, arrhythmias), or increase in circulating markers consistent with cumulative myocardial damage (TnI or CRP).

### 4.5. Cardiac Magnetic Resonance Follow-Up

CMR exams were acquired to evaluate the effect of the administered therapies. Studies were performed one week after infarct induction immediately before therapy administration and 10 weeks later, prior to PES and euthanasia, following a previously described protocol [29]. In brief, anesthetized pigs were positioned in sternal decubitus inside a 1.5T MRI system (Intera 1.5 T, Philips Medical Systems, Best, The Netherlands) and short-axis acquisitions were performed, including breath-hold gradient-echo cine images and breath-hold 3D gradient-echo inversion-recovery sequence 10 min after administering 0.2 mmol/kg of gadobutrol (Gadovist 1.1 mmol/L, Bayer Schering Pharma AG, Berlin, Germany), adjusting myocardial nulling with a previous Look-Locker sequence. Images were processed to calculate infarct size (IS) and to assess end-diastolic volume (LVEDV), end-systolic volume (LVESV), and ejection fraction (LVEF). LVEDV and LVESV were indexed to body surface area (BSA) to allow serial comparison over time. MR images were analyzed by a researcher blinded to the group using commercially available software (Extended MR WorkSpace, Philips Medical Systems, Best, The Netherlands). For these analyses, manual delineation of the endocardial and epicardial borders in end-diastolic and end-systolic cine short-axis views was performed in all slices; then, we calculated LVEDV, LVESV, and LVEF. Similarly, IS was calculated from the delayed enhancement images by manually defining the normal and infarcted myocardium with computer assistance to obtain the percentage of infarcted left ventricle. The papillary muscles were not included. Central dark zones within the area of hyperenhancement were included.

### 4.6. End Study

Ten weeks after infarct induction, a last CMR was obtained, followed by PES and coronariogram to reassess flow in the LAD. PES was carried out by means of a quadrapolar catheter (Marinr SC Steerable Quadrapolar Catheter, Medtronic, Minneapolis, MN, USA) inserted sequentially into the left and right ventricles to analyze the inducibility of arrhythmias. PES was performed at 3 different cycle lengths with up to 4 extra stimuli, with coupling intervals decreasing in 10 ms until S2 reached the refractory period or a minimum coupling interval of 200 ms. Long-term safety was determined in terms of sudden cardiac death and arrhythmia inducibility on the programmed electrical stimulation at end-study day.

Once the follow-up was completed, animals were euthanized by a lethal dose of potassium chloride (1–2 mmol/kg) while under deep anesthesia, as recommended by the relevant authorities.

### 4.7. Postmortem Studies

Immediately after euthanasia, hearts were harvested and weighed, and pictures were taken to document infarct location. The hearts were then sliced in 10–15 mm slices, one of which provided samples for proteomic analyses: 1–2 g of tissue from the infarcted and border zone were collected within minutes of euthanasia, frozen in liquid nitrogen, and cryopreserved at –80 °C for later proteomic analysis.

Sliced hearts were submerged in 4% formalin for a minimum of 48 h and then processed for histopathology. Sections were obtained from ischemic, remote, and border zones in all animals and stained with HE and MT to detect and characterize inflammation, fibrosis, and the existence of any abnormal tissue growth. Fibrotic changes were evaluated further using picrosirius red staining under polarized light microscopy and by determining total collagen, collagen I, III, and the I/III ratio in the border and infarct core zones.

### 4.8. Proteomics

High-throughput quantitative proteomic analysis of infarcted tissue was performed in a subset of animals (n = 5 in CON group and n = 6 in D1 and D3 groups) to identify BP based on the protein abundance changes between groups. Proteins were extracted from tissue samples as previously described [59], and protein concentration in the resulting preparations was determined using the RCDC Protein Assay Kit (Bio-Rad, Hercules, CA, USA). Protein tryptic digestion was carried out using Nanosep 30 K Omega filters (Pall Life Sciences, Westborough, MA, USA), as previously described [60], after which the resulting peptides were isobaric-labeled with 16-plex reporter ions and with additional 18-plex reporter ions using Tandem Mass Tags (TMT, Thermo Scientific, Waltham, MA USA) reagents, following the manufacturer’s instructions. Liquid chromatography tandem mass spectrometry (LC-MS/MS) analysis was performed on an Easy-nLC 1000 HPLC system (Thermo Fisher Scientific) coupled via a nanoelectrospray ion source (Thermo Fisher Scientific) to an Orbitrap Fusion mass spectrometer (Thermo Fisher Scientific). Briefly, labelled peptides were loaded in buffer A (0.1% of formic acid in water (*v*/*v*)) onto a C-18 reversed phase nano-column (75 μm I.D. and 50 cm, Acclaim PepMap) and separated in a continuous acetonitrile gradient consisting of 8–28% B-solution (B = 0.1% formic acid (*v*/*v*) in acetonitrile) for 300 min and at a flow rate of ~200 nL/min. Mass spectra were acquired in a data-dependent manner with a 3 s TopSpeed method in the Orbitrap analyzer, with a 400–1500 m/z range and 60,000 FT resolution. HCD fragmentation was performed at 36 normalized collision energy, and MS/MS spectra were analyzed at 30,000 resolution in the Orbitrap. For peptide identification, the raw LC-MS/MS data were searched using the SEQUEST HT algorithm implemented in Proteome Discoverer 2.5 (Thermo Scientific) [61] against a UniProtKB [62] database comprising human and pig protein sequences (November 2023) concatenated with decoy sequences generated using DecoyPyrat (version 1.5.0) [63]. The false discovery rate (FDR) was calculated using the corrected Xcorr score (cXcorr) [64] and the target/decoy competition strategy applying the picked FDR method at the peptide level [65], with an additional filter for precursor mass tolerance of 15 ppm [66]. A 1% FDR was employed as the criterion for peptide identification. The quantitative information was extracted from the TMT reporter intensity in the raw LC-MS/MS data, and the quantification of protein abundances changes across myocardial samples was carried out on the basis of the WSPP model [67] and the generic integration algorithm (GIA) [68] using iSanXoT software package (version 2.1.1) [69]. The Limma package (version 3.56.0) [70] was used to ascertain statistical significance by means of *p*-values, allowing for a comprehensive assessment of protein abundance changes associated with one- and three-dose treatments respect to the control group.

The Database for Annotation, Visualization, and Integrated Discovery (DAVID) [71,72] was utilized to perform functional enrichment analysis based on significant alterations in protein abundance quantified via Z-score between one- and three-dose treatments. PPI networks were generated using Cytoscape (v. 3.10.3) with the StringApp plugin. Two distinct network analyses were performed: the first aimed to illustrate the interactions between the most relevant BP within gene ontology (GO) and their associated proteins; the second encompassed all significant protein interactions to identify critical proteins, or hubs, within the interactome by analyzing network topology using CytoHubba (version 0.11.3) [73] with the maximal clique centrality (MCC) algorithm. Finally, a summary table outlining key proteins and their hypothesized roles in post–myocardial infarction repair was generated to facilitate interpretation of the results (Table S1).

### 4.9. Data Analysis

Data are presented as means and standard error of the mean. Normality was checked using the Shapiro–Wilk test, and differences between groups were then compared using one-way ANOVA and Student’s *t*-tests or a Fisher–Freeman–Halton test for inducibility data. All analyses were two-tailed, and a *p* < 0.05 was considered significant.

## Figures and Tables

**Table 1:** Evolution of troponin I and C-reactive protein over study time.

	CON (n = 8)			D1 (n = 8)			D3 (n = 8)						
							1st Dose		2nd Dose		3rd Dose		Final
	PRE	POST	End-Study	PRE	POST	End-Study	PRE	POST	PRE	POST	PRE	POST	End-Study
TnI (µg/L)	0.42 (0.12)	0.41 (0.01)	0.01 (0.0)	0.44 (0.11)	0.43 (0.11)	0.07 (0.07)	0.19 (0.09)	0.33 (0.15)	0.05 (0.02)	0.07 (0.02)	0.03 (0.01)	0.03 (0.01)	0.02 (0.01)
CRP (mg/L)	0.39 (0.12)	0.44 (0.15)	0.39 (0.13)	0.93 (0.38)	0.40 (0.20)	0.55 (0.14)	0.28 (0.10)	0.26 (0.09)	0.66 (0.15)	0.44 (0.19)	0.23 (0.12)	0.14 (0.12)	0.36 (0.15)

PRE: pre-injection. POST: post-injection. TnI: Troponin I. CRP: C-reactive protein. CON: control group. D1: group receiving one CDC dose. D3: group receiving 3 doses of CDCs. Data presented as mean (standard error of the mean). Intra-group (PRE versus POST) comparisons were performed using paired *t*-test. End-study values were compared between groups using one-way ANOVA. *p* > 0.05 (N.S.) in all comparisons.

**Table 2:** Cardiac function.

	CON (n = 8)		D1 (n = 8)		D3 (n = 8)		*p*-Value (10 Weeks)
	1 Week	10 Weeks	1 Week	10 Weeks	1 Week	10 Weeks	
IS (%)	19.5 (2.5)	10.3 (0.8)	17.4 (1.3)	10.1 (1.6)	18.9 (2.4)	12.7 (1.2)	0.34
LVEF (%)	19.0 (1.9)	24.2 (2.1)	23 (2.0)	21.4 (2.8)	21.3 (1.1)	27.5 (4.1)	0.39
LVEDVi (mL/m^2^)	98.4 (5.6)	104.4 (8.0)	103 (5.3)	107.3 (9.0)	81.6 (5.6)	81.6 (7.4)	0.09
LVESVi (mL/m^2^)	79.4 (5.3)	79.3 (7.0)	79.5 (5.3)	85.7 (10.1)	64.1 (4.1)	60.1 (7.9)	0.13

CON: control group. D1: group receiving one CDC dose. D3: froup receiving 3 doses of CDCs. IS: infarct size. LVEF: left ventricular ejection fraction. LVEDVi: left ventricular end-diastolic volume indexed to body surface area. LVESVi: end-systolic volume indexed to body surface area. Infarct area is expressed as % of the left ventricle. Data presented as mean (standard error of the mean). Comparisons between groups were performed using one-way ANOVA.

## Data Availability

The original contributions presented in this study are included in the article/Supplementary Materials. Further inquiries can be directed to the corresponding author. The mass spectrometry proteomics data have been deposited to the ProteomeXchange Consortium via the PRIDE [74] partner repository with the dataset identifier PXD070301.

## Supplementary Materials

The following supporting information can be downloaded at: https://www.mdpi.com/article/10.3390/ijms262311294/s1.

## Abbreviations

Table following abbreviations are used in this manuscript:

MI	Myocardial infarction
CDCs	Cardiosphere-derived cells
CGM	Cardiomyocyte growth medium
LAD	Left anterior descending coronary artery
IR	Ischemia reperfusion
HF	Heart failure
CPCs	Cardiac progenitor cells
AMI	Acute myocardial infarction
CMR	Cardiac magnetic resonance
PES	Programmed electrical stimulation
LW	Large white
LVEF	Left ventricular ejection fraction
TnI	Troponin I
CRP	C-reactive protein
IS	Infarct size
LVEDVi	Left ventricular end-diastolic volume indexed to body surface area
LVESVi	End-systolic volume indexed to body surface area
HE	Hematoxylin eosin
MT	Masson’s trichrome
DAPs	Differentially abundant proteins
BP	Biological process
GO	Gene ontology
PPI	Protein–protein interaction
IC	Intracoronary
MSCs	Mesenchymal stromal cells
S-CDCs	Cardiosphere-derived cell secretomes
PBSC	Peripheral blood stem cell
BM-MNC	Bone marrow mononuclear cell
ECM	Extracellular matrix
ALB	Serum albumin
FAAH	Fatty acid amide hydrolase
BSA	Body surface area
DAVID	Database for Annotation, Visualization, and Integrated Discovery
MCC	Maximal clique centrality
