# Supplementary material for: Administration of Single or Repeated Doses of CDCs in a Swine Model of Reperfused Myocardial Infarction: Magnetic Resonance and Proteomics Evaluation
Source: Int J Mol Sci. 2025 Nov 22;26(23):11294. doi: 10.3390/ijms262311294 (PMC12692557; doi:10.3390/ijms262311294)
Supplement: Supplementary file 1 [file ijms-26-11294-s001.zip › ijms-3998859-supplementary.pdf]

Administration of Single or Repeated Doses of CDCs in a Cardiac

Ischemia/Reperfusion Injury Swine Model: Magnetic Resonance and

Proteomics Evaluation

Maria Angeles de Pedro, Claudia Baez-Diaz, Inmaculada Jorge, Fátima Vázquez-Lopez, Axiel Torrescusa-Bermejo, Beatriz Martinez-Fernandez, María Pulido, Esther López, Jesús Vázquez-Cobos, Francisco M. Sánchez-Margallo, Veronica Crisostomo

Supplementary Figures

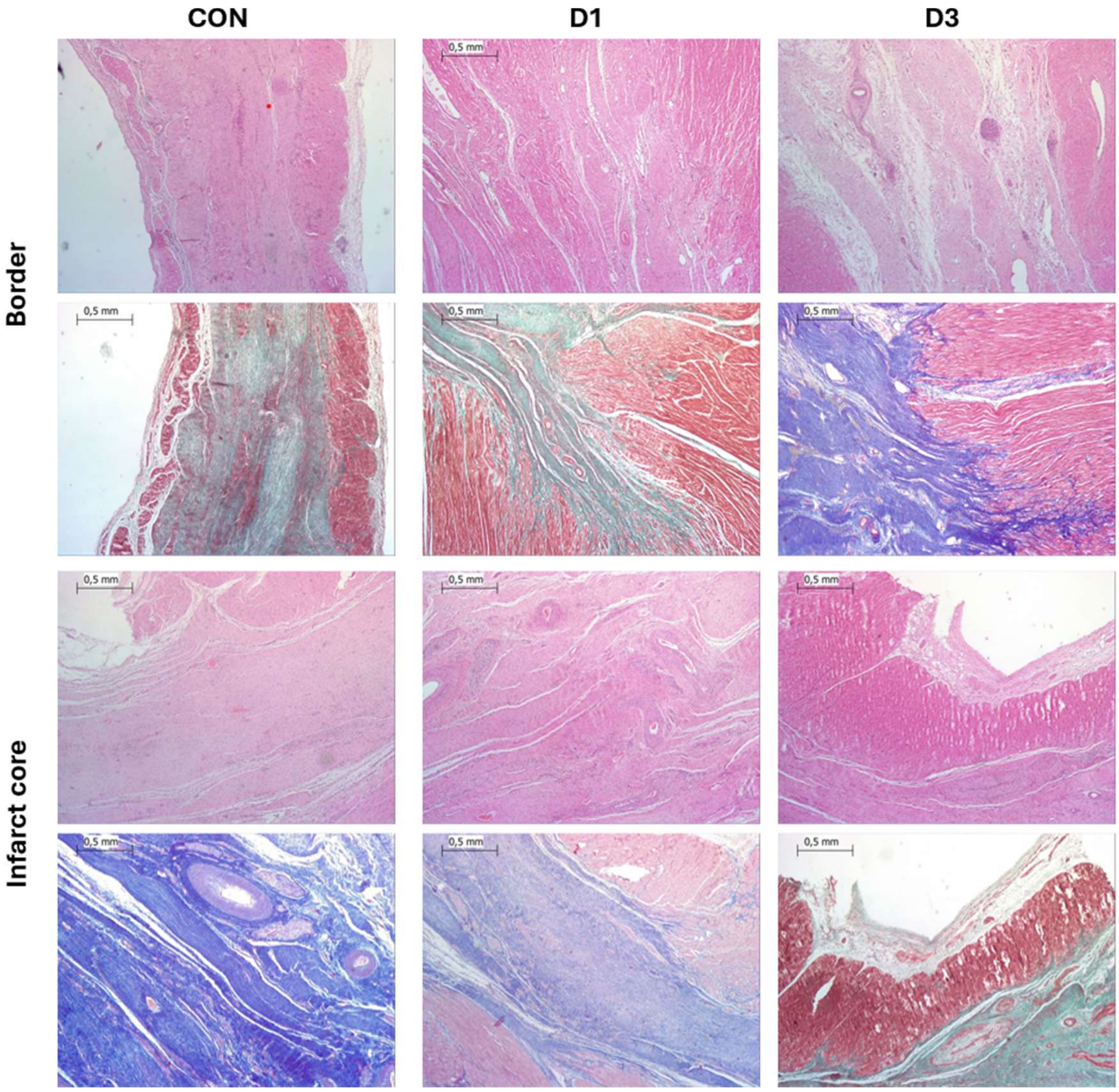

Supplementary Figure S1. Representative microscopy images of Hematoxylin-Eosin and Masson's Trichrome staining in border and infarct core samples from swine belonging to the three experimental groups (CON: Control, D1: one CDCs dose, D3: three CDCs doses).

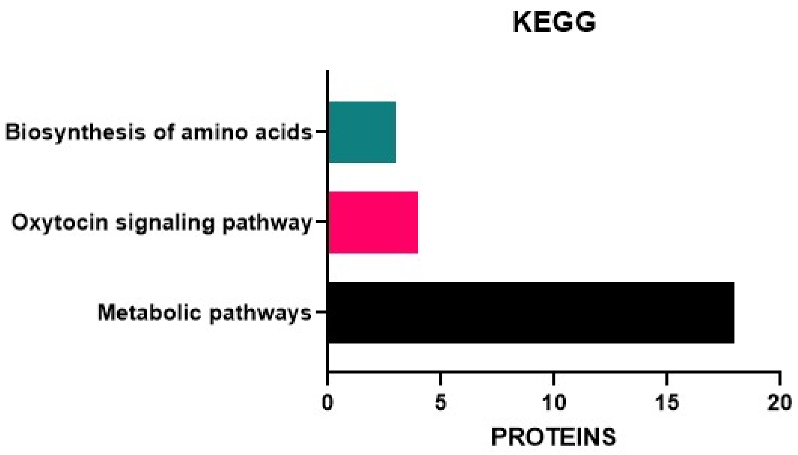

Supplementary Figure S2. KEGG pathway enrichment of differentially abundant proteins (DAPs) after repeated CDC administration.

Supplementary Table

Protein	Gene	Regulation status	Proposed role in MI repair
A0A287B5T1	ARPIN	DOWN	Reduced ARPIN may facilitate fibroblast and endothelial cell migration, supporting angiogenesis and tissue remodeling during late post-MI repair.
F1S762	PODN	UP	Increased PODN may limit excessive fibrosis and support organized ECM remodeling, promoting favorable scar formation and functional recovery during late post-MI repair.
P08138	NGFR	DOWN	Reduced NGFR may decrease apoptosis in cardiomyocytes while potentially limiting reparative neurotrophic and angiogenic signaling during late post-MI remodeling.
P54764	EPHA4	UP	Increased EPHA4 may promote angiogenesis and reparative cell migration, supporting tissue remodeling and improved perfusion during late post-MI repair.
A0A4X1VYT0	GYS1	UP	Increased GYS1 may enhance myocardial glycogen storage, supporting energy supply, cardiomyocyte survival, and functional stability during late post-MI remodeling.
A0A5G2QE35	GBE1	UP	Increased GBE1 may improve glycogen structure and energy availability, supporting cardiomyocyte metabolism and functional stability during late post-MI remodeling.
A0A4X1TS17	AGL	UP	Increased AGL may enhance glycogen mobilization, supporting cardiomyocyte energy supply and functional stability during late post-MI remodeling.

Protein	Gene	Regulation status	Proposed role in MI repair
A0A5G2QV95	RHOD	DOWN	Reduced RHOD may limit excessive cytoskeletal remodeling and maladaptive cell migration, supporting organized tissue repair during late post-MI remodeling.
P49757	NUMB	DOWN	Reduced NUMB may modulate Notch signaling, limiting maladaptive fibroblast activation and fibrosis, thereby supporting organized tissue remodeling during late post-MI repair.
A0A286ZXJ6	PPP3CA	DOWN	Reduced PPP3CA may attenuate pathological hypertrophy and maladaptive remodeling, supporting favorable structural and functional outcomes during late post-MI repair.
A0A5G2R9I6	ANK2	UP	Increased ANK2 may enhance cardiomyocyte calcium handling and structural organization, supporting function, but could also increase arrhythmia risk during late post-MI remodeling.
A0A287ACR4	TNNI3K	UP	Increased TNNI3K may influence cardiomyocyte contractility and survival, but could also exacerbate oxidative stress and adverse remodeling during late post-MI repair.
Q9HAQ2	KIF9	UP	Increased KIF9 may support intracellular transport and structural organization in cardiomyocytes, but could also perturb cytoskeletal dynamics, with uncertain effects on late post-MI remodeling.
P00747	PLG	DOWN	Reduced PLG may limit ECM remodeling and fibrinolysis, potentially promoting stiffer scar formation and maladaptive remodeling during late post-MI repair.
A0A287B960	MYLK3	DOWN	Reduced MYLK3 may decrease cardiomyocyte contractility, potentially impairing cardiac performance, while possibly limiting

Protein	Gene	Regulation status	Proposed role in MI repair
			maladaptive stress responses during late post-MI remodeling.
A0A5G2QIU0	CSFR1	DOWN	Reduced CSF1R may impair macrophage-mediated tissue repair and remodeling, potentially limiting clearance of necrotic tissue and adaptive remodeling during late post-MI repair.
A0A287ABZ9	ALPK3	UP	Increased ALPK3 may support cardiomyocyte structural integrity and proteostasis, promoting functional stability during late post-MI remodeling.
P31152	MAPK4	DOWN	Reduced MAPK4 may limit maladaptive hypertrophy but could also impair cardiomyocyte survival and reparative signaling during late post-MI remodeling.
P02768	ALB	UP	Increased ALB may enhance antioxidant capacity and metabolic support, indirectly supporting cardiomyocyte survival and late post-MI remodeling.
Q9TUI8	FAAH	UP	Increased FAAH may help modulate inflammation and extracellular matrix remodeling, potentially limiting cardiomyocyte loss and maladaptive fibrosis during late post-MI repair.
A0A5G2QXR8	NT5E	DOWN	Reduced NT5E may impair adenosine-mediated anti-inflammatory and reparative signaling, potentially exacerbating fibrosis and adverse remodeling during late post-MI repair.
P00558	PGK1	UP	Increased PGK1 may support energy production and cardiomyocyte survival, but could also indicate ongoing metabolic stress during late post-MI remodeling.
A0A4X1VS57	EEF2	UP	Increased eEF2 may enhance protein synthesis and cardiomyocyte survival, but could also promote apoptosis under stress, reflecting a

Protein	Gene	Regulation status	Proposed role in MI repair
			context-dependent role during late post-MI remodeling.
Q9P2J5	LARS1	UP	Increased LARS1 may support protein synthesis and cardiomyocyte survival via mTOR signaling, but could also contribute to maladaptive hypertrophy during late post-MI remodeling.
A0A287A8M1	IDH1	DOWN	Reduced IDH1 may impair antioxidant defenses, increasing oxidative stress and potentially exacerbating adverse remodeling during late post-MI repair.
A0A286ZLB1	TRAP1	UP	Increased TRAP1 may preserve mitochondrial function and reduce oxidative stress, promoting cardiomyocyte survival during late post-MI remodeling.
A0A286ZST2	PDCD11	UP	Increased PDCD11 may support cardiomyocyte survival and protein synthesis, but could also affect apoptotic regulation during late post-MI remodeling.
F1RW04	TRIM65	DOWN	Reduced TRIM65 may impair autophagy and mitochondrial function, enhancing inflammasome activation, apoptosis, and fibrosis, thereby promoting adverse remodeling during late post-MI repair.

Supplementary Table S1. Summary of key proteins and their hypothesized roles in post-MI repair. Regulation status indicates upregulation (up) or downregulation (down) in three CDC doses (D3) versus a single dose (D1).

Supplementary methodology

Proteomics analysis

Preparation of protein extracts and tryptic digestion

Myocardial tissues were excised from experimental animals immediately after sacrifice, snap-frozen in liquid nitrogen, and stored at -80°C until processing. Proteins extracts from homogenized tissue were obtained using ceramic beads (MagNa Lyser Green Beads apparatus, Roche, Germany) in extraction buffer (50 mM Tris-HCl, 1 mM EDTA, 1.5% SDS, 50 mM iodoacetamide, pH 8.5), as previously described [1]. Protein concentration in the resulting preparations was determined using the RCDC Protein Assay Kit (Bio-Rad, Hercules, CA, USA). We applied the filter-based FASILOX technology [2]. Briefly, 200 μg of protein extract was diluted in urea sample solution (8 M urea in 100mM Tris–HCl, pH 8.5) and loaded on a Nanosep 30 K Omega filters (Pall Life Sciences, MA, USA) .After centrifugation and a wash step with the same buffer, protein thiol groups were reduced with 50 mM dithiothreitol and then alkylated using 50 mM methyl methanethiosulfonate. Protein digestion was carried out overnight at 37°C with sequencing grade trypsin (Promega, Madison, WI, USA) at 1:40 (w/w) trypsin:protein ratio in digestion buffer (50 mM ammonium bicarbonate, pH 8.5), after which the resulting tryptic peptides were recovered by centrifugation. Trifluoroacetic acid (TFA) was added to a final concentration of 1% and the peptides were desalted on C18 Oasis HLB extraction cartridges (Waters Corporation, Milford, MA, USA) and dried-down.

Peptide isobaric labelling

The peptides were subjected to multiplexed isobaric labeling (TMT18plex). The TMT18plex batch was used to label 17 individuals' samples and one channel was reserved for reference internal standard sample created by pooling the samples from Control group. Briefly, the peptide samples were taken up in 1 M triethylamonium bicarbonate and their concentration was determined using a Direct Detect infrared spectrometer (Millipore, Billerica, MA, USA). Equal amounts of the resulting peptides were isobarically labelled with 16-plex reporter ions and with additional 18-plex reporter ions Tandem Mass Tags (TMT, Thermo Scientific, San Jose, MA, USA) reagents,

following the manufacturer's instructions. The labeled peptides samples were mixed appropriately and dried-down.

High pH reversed-phase fractionation

Aliquots of the dried labeled peptide samples were taken up in 300 μL of 0.1% TFA and separated into five fractions using the high pH reversed-phase peptide fractionation kit (Thermo Scientific). The spin column was equilibrated with 300 μL of acetonitrile (ACN) twice, followed by 2 x 300 μL of 0.1% TFA. Then the samples were loaded onto the columns and centrifuged at 3,000 x g for 2 minutes. The columns were then washed with 300 μL of water. The bound peptides were eluted into five fractions with 300 μL of freshly prepared elution solutions: (1) 12.5% acetonitrile (ACN), 87.5% triethylamine; (2) 15% ACN, 85% triethylamine; (3) 17.5% ACN, 82.5% triethylamine; (4) 20% ACN, 80% triethylamine; and (5) 50% ACN, 50% triethylamine. The so-obtained fractions were dried and stored at −20°C until MS analysis.

Liquid chromatography tandem mass spectrometry analysis (LC-MS/MS)

LC-MS/MS analysis was performed using an Easy nanoLC 1000 (ThermoFisher Scientific) coupled to an Orbitrap Fusion Trihybrid Mass Spectrometer (Thermo Fisher Scientific) using an Acclaim PepMap 100 C18 2 cm x 75 μm internal diameter as trapping column (Thermo Fisher Scientific) and a PepMap RSLC C18 EASY-Spray column 50 cm x 75 μm internal diameter as analytical column (Thermo Fisher Scientific). Peptides were loaded in 0.1% formic acid in water (v/v) buffer and eluted in a continuous acetonitrile gradient consisting of 8-28% B-solution (B=0.1% formic acid (v/v) in acetonitrile) for 300 min, at a flow rate of ~200 nL/min. Mass spectra were acquired in a data-dependent manner, with an automatic switch between MS and MS/MS with a 3 s-TopSpeed method and 40 s dynamic exclusion. MS spectra were acquired in the Orbitrap analyzer using full ion-scan mode with a 400-1500 m/z range and 60,000 FT resolution. The automatic gain control target was set at 2×10^5 with a 50 ms maximum injection time. HCD fragmentation was performed at 36% of normalized collision energy and MS/MS spectra were analyzed at a 30,000 resolution in the Orbitrap with the automatic gain control target set at 5×10^5 and 120 ms maximum injection time.

Peptide and protein identification

For peptide identification, the raw LC-MS/MS data were searched using the SEQUEST HT algorithm implemented in Proteome Discoverer 2.5 (Thermo Scientific) [3] against a UniprotKB [4] database comprising human and pig protein sequences (Nov 2023) concatenated with decoy sequences generated using DecoyPyrat [5]. Trypsin digestion was set with a maximum of two missed cleavages. Cys carbamidomethylation (57.021 Da), Cys methylthiolation (45.988 Da), and Met oxidation (15.995 Da) were set as dynamic modifications, and TMT labeling at the N-terminal end and at Lys (304.207 Da) were set as fixed modifications. Precursor mass tolerance was set at 800 ppm, fragment mass tolerance at 0.02 Da and precursor charge range to 2-4. The false discovery rate (FDR) was calculated using the corrected Xcorr score (cXcorr) [6] and the target/decoy competition strategy applying the picked FDR method at the peptide level [7], with an additional filter for precursor mass tolerance of 15 ppm [8]. A 1% FDR was employed as the criterion for peptide identification. Peptide-to-protein assignment was carried out using a method developed in-house, where each peptide is assigned to the most likely protein. Firstly, proteins are ranked according to the number of peptides with which they are identified. Then the algorithm assigns to each peptide the protein with the highest number of peptides. In cases of tied proteins, it prioritizes assignments based on the number of peptide-spectrum matches, with persistent ties resolved using user-defined regular expression "*Sus scrofa*" on the protein FASTA header.

Protein and functional category quantification

The quantitative information derived from the TMT reporter intensity in the raw LC-MS/MS data was integrated from the spectrum level to the peptide level and subsequently to the protein level, according to the WSPP model [9] and the Generic Integration Algorithm (GIA)[10], using iSanXoT software package[11]. In this model, quantitative protein values are expressed using the standardized variable Zq (i.e., normalized log2-ratios expressed in units of standard deviation according to the estimated variances). The Limma package [12] was used to ascertain statistical significance by means of p-values.

Supplementary references

1. Binek, A.; Castans, C.; Jorge, I.; Bagwan, N.; Rodríguez, J.M.; Fernández-Jiménez, R.; Galán-Arriola, C.; Oliver, E.; Gómez, M.; Clemente-Moragón, A.; et al. Oxidative Post-translational Protein Modifications upon Ischemia/Reperfusion Injury. *Antioxidants* **2024**, *13*, 106.
2. Bonzon-Kulichenko, E.; Camafeita, E.; López, J.A.; Gómez-Serrano, M.; Jorge, I.; Calvo, E.; Núñez, E.; Trevisan-Herraz, M.; Bagwan, N.; Bárcena, J.A.; et al. Improved integrative analysis of the thiol redox proteome using filter-aided sample preparation. *Journal of Proteomics* **2020**, *214*, 103624, doi:https://doi.org/10.1016/j.jprot.2019.103624.
3. Orsburn, B.C. Proteome Discoverer—A Community Enhanced Data Processing Suite for Protein Informatics. *Proteomes* **2021**, *9*, 15.
4. Consortium, T.U. UniProt: the Universal Protein Knowledgebase in 2023. *Nucleic Acids Research* **2022**, *51*, D523-D531, doi:10.1093/nar/gkac1052.
5. Wright, J.C.; Choudhary, J.S. DecoyPyrat: Fast Non-redundant Hybrid Decoy Sequence Generation for Large Scale Proteomics. *J Proteomics Bioinform* **2016**, *9*, 176-180, doi:10.4172/jpb.1000404.
6. Keller, A.; Nesvizhskii, A.I.; Kolker, E.; Aebersold, R. Empirical Statistical Model To Estimate the Accuracy of Peptide Identifications Made by MS/MS and Database Search. *Analytical Chemistry* **2002**, *74*, 5383-5392, doi:10.1021/ac025747h.
7. Prieto, G.; Vázquez, J. Protein Probability Model for High-Throughput Protein Identification by Mass Spectrometry-Based Proteomics. *Journal of Proteome Research* **2020**, *19*, 1285-1297, doi:10.1021/acs.jproteome.9b00819.
8. Bonzon-Kulichenko, E.; Garcia-Marques, F.; Trevisan-Herraz, M.; Vázquez, J. Revisiting Peptide Identification by High-Accuracy Mass Spectrometry: Problems Associated with the Use of Narrow Mass Precursor Windows. *Journal of Proteome Research* **2015**, *14*, 700-710, doi:10.1021/pr5007284.
9. Navarro, P.; Trevisan-Herraz, M.; Bonzon-Kulichenko, E.; Núñez, E.; Martínez-Acedo, P.; Pérez-Hernández, D.; Jorge, I.; Mesa, R.; Calvo, E.; Carrascal, M.; et al. General Statistical Framework for Quantitative Proteomics by Stable Isotope Labeling. *Journal of Proteome Research* **2014**, *13*, 1234-1247, doi:10.1021/pr4006958.
10. García-Marqués, F.; Trevisan-Herraz, M.; Martínez-Martínez, S.; Camafeita, E.; Jorge, I.; Lopez, J.A.; Méndez-Barbero, N.; Méndez-Ferrer, S.; del Pozo, M.A.; Ibáñez, B.; et al. A Novel Systems-Biology Algorithm for the Analysis of Coordinated Protein Responses Using Quantitative Proteomics *. *Molecular & Cellular Proteomics* **2016**, *15*, 1740-1760, doi:10.1074/mcp.M115.055905.
11. Rodríguez, J.M.; Jorge, I.; Martinez-Val, A.; Barrero-Rodríguez, R.; Magni, R.; Núñez, E.; Laguillo, A.; Devesa, C.A.; López, J.A.; Camafeita, E.; et al. iSanXoT: A standalone application for the integrative analysis of mass spectrometry-based quantitative proteomics data. *Computational and Structural Biotechnology Journal* **2024**, *23*, 452-459, doi:10.1016/j.csbj.2023.12.034.
12. Ritchie, M.E.; Phipson, B.; Wu, D.; Hu, Y.; Law, C.W.; Shi, W.; Smyth, G.K. limma powers differential expression analyses for RNA-sequencing and microarray studies. *Nucleic Acids Research* **2015**, *43*, e47-e47, doi:10.1093/nar/gkv007.
